# Shape of the Doppler Sonographic Systolic Blood Flow Profile of the Pulmonary Artery of Healthy Racing Pigeons and the Influence of Anesthesia

**DOI:** 10.3390/vetsci11120679

**Published:** 2024-12-23

**Authors:** Carolin Girard, Lajos Koy, Norbert Kummerfeld, Michael Pees, Michael Fehr, Marko Legler

**Affiliations:** Department of Small Mammal, Reptile and Avian Medicine and Surgery, University of Veterinary Medicine Hannover, Bünteweg 9, 30559 Hanover, Germany

**Keywords:** sonography, systolic blood flow, acceleration time, deceleration time, acceleration phase, deceleration phase

## Abstract

There is scant information available about the blood flow of the pulmonary artery in avian cardiology. In human medicine, the shape of the Doppler sonographic blood flow profile of the pulmonary artery can be used to access the pressure conditions of the right heart. With this background, this study focused on the examination of the acceleration and deceleration phase of the pulsed-wave Doppler flow profile of the pulmonary artery of healthy racing pigeons. The results showed a significant difference between the pulmonary artery and aorta. The Doppler flow profile of the aorta was characterized by a short acceleration phase; however, the pulmonary flow profile revealed an acceleration similar to that of the deceleration phase. Neither profile changed considerably under the influence of heart rate changes and anesthesia with isoflurane. A negative correlation of the pulmonary acceleration phase with the left diastolic A wave and the systolic pulmonary blood flow velocities could be found. This indicates the influence of pressure changes in the heart on the flow profiles. The results of this study allow for the use of the shape of the Doppler sonographic blood flow profile of the pulmonary artery in the assessment of cardiovascular diseases in avian medicine, especially in racing pigeons.

## Simple Summary

The evaluation of Doppler sonographic images of healthy racing pigeons, with a focus on the systolic blood flow of the pulmonary artery, revealed significant differences between the systolic Doppler sonographic blood flow profiles of the pulmonary artery and the aorta. The pulmonary blood flow profile was characterized by a round shape, with a similar acceleration and deceleration phase. In contrast to this, the blood flow profile of the aorta showed a short acceleration and a long deceleration phase. Neither profile changed considerably under the influence of heart rate and anesthesia. Comparable to the methods used in small mammal and human medicine, these results indicate that, in avian medicine, it also seems possible to assess the pressure conditions in the heart and to recognize changes in the right heart pressure due to heart disease using the blood flow profile of the pulmonary artery.

## 1. Introduction

The blood flow of the aorta (AO) and pulmonary artery (PA) differs significantly, as has been specifically studied in small animal and human medicine [1,2,3,4]. The lower pressure system of the right heart and the pulmonary circulation lead to a pulmonary artery Doppler flow profile (PAFP) with a comparable acceleration and deceleration phase, which results in a near-isosceles triangular shape [1]. The Doppler flow profile of the aorta (AOFP) shows a very short acceleration time (AT) and a longer deceleration time (DT), caused by the higher pressure system of the body circulation and the left heart [1].

In birds, there is only scant information about the blood flow of the pulmonary artery. Initial studies in birds showed a PAFP similar to that of healthy mammals, with an AT that is comparable to the DT, which results in a rounded appearance of this blood flow profile in birds. The AOFP of birds appears similar to the AOFP of mammals, with a very short AT [5,6].

Studies in small animal and human medicine have shown that diseases that lead to pulmonary hypertension have a significant influence on the shape of the PAFP. The higher pressure in these diseased animals and humans leads to a PAFP similar to an AOFP [7,8,9,10,11]. An initial study on Congo Grey parrots (*Psittacus erithacus*) with left and right heart failure showed that in the diseased groups, the AT was also significantly shorter, and the shape of the PAFP approached the AOFP, in some cases [12]. This means that the PAFP parameter can also probably be used to diagnose pulmonary hypertension in birds. However, there is little information on the correlation of the profile with diastolic and systolic blood flow velocities in the heart and the influence of heart rate and anesthesia with isoflurane on these systolic profiles in healthy birds [5,6,13,14,15,16].

With this background, the aim of this study was to examine the shape of the PAFP and AOFP in detail and to determine the possible influences of different physiological parameters on the flow profiles of healthy racing pigeons (*Columba livia* f. domestica).

## 2. Materials and Methods

The Doppler sonographic images of ultrasonographically examined healthy racing pigeons were evaluated. The sonographic study on the pigeons was conducted following the German animal welfare regulations and with the permission of the relevant German authorities (Niedersächsisches Landesamt für Verbraucherschutz und Lebensmittelsicherheit, protocol code: 33.12-42502-04-15/1864).

### 2.1. Examined Racing Pigeons

In this study, Doppler sonographic blood flow profiles of the hearts of racing pigeons (*Columba livia* f. dom.; *n* = 43), including both males (n = 16) and females (n = 27), were evaluated. The pigeons were trained for racing and were 2.3 years ± SD 1.7 (range: 0.5 to 8 years) old, with a body mass of 468.2 g ± SD 51.64 (range: 352–577 g). Their sternal length, measured from the visible sternocoracoid joint to the end of the sternum in laterolateral radiographic images, was 73.4 mm ± SD 3.1 mm (range: 63.2–81.0 mm). The pigeons were housed in indoor aviaries and were provided with a commercial pigeon seed mix and fresh drinking water ad libitum. The owner routinely vaccinated the pigeons for pigeon avian orthoavulavirus 1 (paramyxovirus 1) and salmonellosis. All pigeons exhibited normal feeding and drinking behaviors. Before the ultrasound examinations, the pigeons were acclimated in the new aviaries for two weeks. During this period, bacteriological and parasitological tests of a composite fecal sample of the pigeons for salmonella and endoparasites of the intestine were negative. A microscopic examination of fresh crop samples revealed a low-grade infestation with *Trichomonas gallinae* in some pigeons, and all pigeons were treated with 10 mg of carnidazole once (Spatrix^®^, Elanco Deutschland GmbH, Bad Homburg, Germany). Clinical, hematological, and radiological examinations of the pigeons were unremarkable; X-rays were performed in a laterolateral and dorsoventral position (GIERTH HF400A, GIERTH X-ray International GmbH, Riesa, Germany). The maximum width of the cardiac silhouette (heart score) of the pigeons measured in the ventrodorsal radiograph was 58.8% ± 3.3 (50.3–65.1%) of the maximum width of the thorax. The pigeons’ hematocrit (44.9% ± 1.8; 42.0–49.0%) and the buffy coat (˂1%) levels were both within the reference range for healthy, normally hydrated pigeons [17].

Egg-laying pigeons were excluded from the sonographic examinations to avoid the effects of high coelomic pressure.

### 2.2. Doppler Sonographic Examination

Echocardiographic images were obtained using a 10 MHz phased-array transducer (GE 10S-RS Probe; B Mode 4.5–11.5 MHz; GE VINGMED ULTRASOUND A/S, Horten, Norway) paired with a digital ultrasound system (Vivid 7 Dimension BT08; GE VINGMED ULTRASOUND A/S, Horten, Norway), along with an electrocardiogram (ECG; GE VINGMED ULTRASOUND A/S, Horten, Norway). The ECG was recorded using clamps without teeth placed on the right and left patagium and the left flank fold, following the Einthoven method [18]. For the sonographic examination, the pigeons were fixed in a semi-upright position and held in place by an assistant.

The right parasternal longitudinal horizontal view (“five-chamber view”) was used to assess the Doppler blood flow profile of the pulmonary artery and the atrioventricular valves, while the left parasternal longitudinal horizontal view was used to examine the aortic blood flow [5,19,20]. Pulsed-wave (PW) Doppler sonography with a 1.5 mm sample volume was selected to analyze the blood flow. A wall filter setting of 3.4 cm/s was applied to remove motion from the cardiac structures. The angle correction function was used in each image individually during the measurements.

### 2.3. Anesthesia

Each racing pigeon underwent two echocardiographic examinations, once while awake and once under general anesthesia with isoflurane (Isofluran CP^®^, CP Pharma GmbH, Burgdorf, Germany), with at least a two-day interval between them. Prior to anesthesia, food was withdrawn for four hours and water for half an hour. Anesthesia was induced with 4 vol.% isoflurane in 1 L/min oxygen and maintained with 1 to 3 vol.% (2.2 ± 0.5 vol.%) isoflurane in 1 L/min oxygen using an anesthetic mask (Veterinary mask No. 2, Eickemeyer, Tuttlingen, Germany) and spontaneous breathing in a semi-open anesthesia system (Eickemeyer, Tuttlingen, Germany). The depth of anesthesia was monitored by assessing the toe pinch and wing twitch reflexes. The echocardiographic examination was performed during the surgical anesthesia state, characterized by the absence of the mentioned reflexes and deep, regular breathing cycles. During the examinations, the respiratory rate in awake pigeons was 44.9 ± 8.6 breaths per minute^−1^ (32–60 breaths per minute^−1^) and decreased during anesthesia to 25.4 ± 6.9 breaths per minute^−1^ (14–48 breaths per minute^−1^). Anesthesia and examination time averaged 27.2 ± 7.4 min (14–48 min), following a five-minute induction period. To prevent rapid cooling, the pigeons were placed on a heat mat set to 37°.

### 2.4. Measurements of the Doppler Blood Flow Profile

Different sonographic parameters were measured. The abbreviations are explained in Table 1. The systolic and diastolic peak flow velocities in meters per second (m/s) were evaluated. The heart rate at the time of the measurements was noted and considered when comparing the different velocities. The blood flow time (ejection time; ET) in milliseconds (ms) was calculated from the onset to the termination of flow. The shape of the blood flow profiles of the aorta and pulmonary artery were quantified by the determination of the acceleration time (AT) from the onset of the ejection to the peak flow velocity and the deceleration time (DT) from the peak flow velocity to the end of the blood flow cycle in ms. The percentage of AT and DT on the ET was determined to quantify the acceleration phase (AP) and deceleration phase (DP) and thus, to quantify the flow profiles. The AP and DP were used for further correlation analyses. All measurements were performed over six sequential individual heart beats, and the mean was used for further evaluations. The evaluations were performed in awake and anesthetized pigeons in the same way.

### 2.5. Statistical Analysis

Statistical tests were performed using SPSS^®^ Statistics 28 (IBM, Armonk, NY, USA). Mean, median, standard deviation (SD), and range (Xmin to Xmax) were calculated for the systolic and diastolic parameters of the Doppler flow profiles. The Kolmogorov–Smirnov test showed that some parameters were not normally distributed. The differences between the systolic peak flow velocities and the different parameters of the blood flow profiles of the aorta and the pulmonary artery were calculated using the Mann–Whitney U test because rapidly changing heart rates do not enable useful value pairs to be created between different images. The influence of the anesthesia was calculated with the Wilcoxon signed-rank test. The Spearman’s rank correlation coefficient (r) was used to visualize the influence of the heart rate, age, body mass, and heart score on the aortic and pulmonary blood flow pattern parameters and to show correlations with the blood flow velocities. A level of *p* ≤ 0.05 was assumed for significance. A level of 0.1 < 0.3 was considered a low correlation, 0.3 < 0.5 a moderate correlation, and 0.5 < 0.7 a strong correlation.

## 3. Results

### 3.1. Shape of PAFP and AOFP

**Figure 1:**
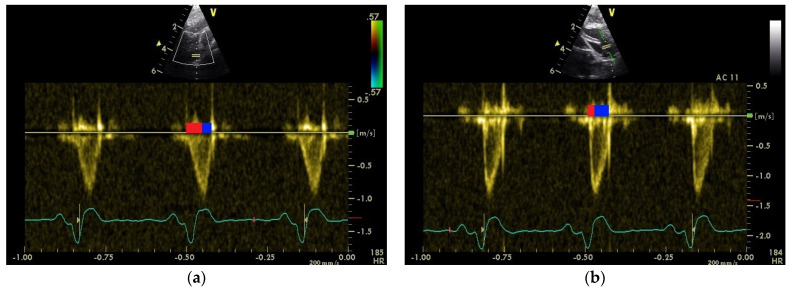
PW Doppler sonographic blood flow profile of the pulmonary artery (**a**) and the aorta (**b**). The acceleration phase of the blood flow is marked with a red line, and the deceleration phase with a blue line.

The shape of the PAFP and the AOFP could be assessed in all 43 pigeons (Figure 1). Because of rapidly changing heart frequencies, in total, 63 PW Doppler flow images of the AO and 60 images of the PA in awake pigeons, and 45 images of the AO and 46 images of the PA in the anesthetized pigeons were examined. An overview of the parameters of the blood flow profiles and the systolic and diastolic blood flow parameters for which a correlation with the shape of the blood flow was tested is shown in Table 2. The heart rates at the time of the measurement have also been added because they have to be considered when comparing the parameters (Table 2).

The examinations showed a significant difference between the shape of the Doppler flow profile of AO and PA (Figure 1). The percentage of the AT of the total ET of the AOFP and PAFP, as an expression of the shape of the blood flow profile, showed that the acceleration phase (AP) of PAFP is significantly longer, and the deceleration phase (DP) of the blood flow of the PA is significantly shorter compared to the AOFP (*p* < 0.001; Mann–Whitney U test) of pigeons (Table 2). The PAFP had a round shape, with an AP significantly longer than in the deceleration phase (*p* < 0.05; Mann–Whitney U test) in awake pigeons. The AOFP was characterized of a short AP and a longer DP (*p* = 0.005; Mann–Whitney U test). As previously described, the aortic ET was significantly shorter than the systolic flow time of the PA in awake and anesthetized pigeons (*p* ≤ 0.001; Mann–Whitney U test [5,6]).

### 3.2. Influence of the Heart Rate on the Shape of the PAFP and AOFP

The heart rate in awake pigeons had a significant negative influence on the ET, AT, and DT of the PA and the AO, as shown in Table 3 (*p* ≤ 0.05). However, the shape of the blood flow of the PA was also influenced by the heart rate in healthy pigeons. There was a moderate significant positive correlation of the AP (r = 0.31; *p* = 0.02) and a strong significant negative correlation of the DP (r = −0.55; *p* = 0.02) of the PAFP with the heart rate in the range of 136–360 heart beats per min (Table 3).

In contrast, there was no significant correlation of the AOFP with the heart rate (*p* = 0.30) in the range of 118–330 beats per min (Table 2). However, a non-significant low negative correlation of the AP (r = −0.13) of the AOFP and a low positive correlation of the DP (r = 0.13) could also be observed.

### 3.3. Influence of Anaesthesia with Isoflurane on the Shape of the PAFP and AOFP

Anesthesia had a significant influence on different sonographic parameters (Table 4). Specifically, the active diastolic and systolic blood flow velocities are significantly affected [6,21]. Regarding the systolic blood flow profiles, the blood flow time and the AT and DT of the AO and PA should be particularly emphasized. The ET was prolonged compared to that noted for awake pigeons (*p* < 0.001; Wilcoxon signed-rank test), as previously described [6]. The AT and the DT of the PA and AO were also significantly extended (*p* < 0.001; Wilcoxon signed-rank test [6]).

However, in contrast to the results for the AOFP (*p* = 0.84; Wilcoxon signed-rank test), anesthesia had a significant influence on the shape of the blood flow of the PA. The AP was significantly shorter in awake pigeons (53.7 to 50.2%; *p* = 0.04; Wilcoxon signed-rank test), and the DP was significantly longer (46.7% to 49.8%; *p* = 0.05; Wilcoxon signed-rank test). This altered PAFP means that the AT does not differ significantly from the DT of the PAFP (*p* = 0.84; Wilcoxon signed-rank test).

Furthermore, under anesthesia, there was an influence of the heart rate on systolic blood flow patterns in pigeons (Table 3). For higher heart rates, the AOFP shows a moderate statistically significant positive longer AP (r = 0.3, *p* = 0.04) and a moderate, non-significant negative shorter DP (r = −0.3; *p* = 0.4). The same blood flow profile changes were noted in the PA, but these were non-significant and displayed a low correlation (AP: r = 0.20; *p* = 0.15; DP: r = −0.2; *p* = 0.15).

### 3.4. Correlation of the Blood Flow Velocities with the Shape of the PAFP and AOFP

In awake pigeons, we found a significant correlation between the active left ventricular filling (A wave velocity left) and the shape of the PAFP (Table 5). Higher A wave velocities of the left ventricle are moderately statistically significantly correlated with a shorter AP (r = −0.37; *p* = 0.03) and a longer DP (r = 0.37; *p* = 0.03) of the PAFP. A moderate significant correlation between the PAFP and the left E-to-A wave ratio (AP: r = 0.37; *p* = 0.03; DP: r = −0.38; *p* = 0.02) in awake pigeons was also observed.

There was also a non-significant low correlation of the peak flow velocities of the PA and the shape of the pulmonary blood flow. Higher velocities in the PA were not significantly lowly correlated with a longer AP (r = 0.22; *p* = 0.09) and a shorter DP (r = −0.22; *p* = 0.09) of the PAFP (Table 5). The peak flow velocities of the PA were significantly lowly (AT) and moderately (DT) negatively correlated with the AT (r = −0.28; *p* = 0.03) and the DT (r = −0.42; *p* = 0.001). In awake pigeons, the shape of the AOFP was not significantly correlated with diastolic and systolic blood flow velocities. However, a non-significant low negative correlation was found with the left E-to-A wave ratio (AP: r = −0.19; *p* = 0.25; DP: r = 0.19; *p* = 0.25).

In anesthetized pigeons, there was a significant moderate correlation between the peak flow velocities of the PA and the shape of the PAFP (Table 6). Higher velocities are correlated moderately and statistically significant negatively with a shorter AP (r = −0.32; *p* = 0.03) and a longer DP (r = −0.32; *p* = 0.03). Moreover, there was a non-significant correlation between the right diastolic blood flow velocities and the shape of the PAFP. The AP was lowly to moderately negatively correlated with the right E and A wave velocities (E_righ_t: r = −0.32; *p* = 0.09; A_right_: r = −0.27; *p* = 0.13), and the DP was lowly to moderately positively correlated with the right diastolic blood flow velocities (E_right_: r = 0.31; *p* = 0.1; A_right_: r = 0.28; *p* = 0.11). There was also a low correlation with the left A wave velocities (AP: r = −0.23; *p* = 0.22; DP: r = 0.22; *p* = 0.22).

The shape of the AOFP under anesthesia is mainly non-significantly and lowly correlated with the peak flow velocities of the AO (AP: r = −0.28; *p* = 0.06; DP: r = 0.28; *p* = 0.06).

### 3.5. Correlation of Pulmonary and Aortic Blood Flow Pattern with Weight, Age, Sex, and Heart Score

There were no significant correlations between the shape of the PAFP and AOFP with the age and the sex of the pigeons.

The shape of the PAFP was significantly moderately correlated with the body mass under anesthesia (AP: r = 0.33; *p* = 0.03; DP: r = −0.32; *p* = 0.03) and the heart score in awake and anesthetized pigeons (AP: r = 0.33; *p* = 0.02; DP: r = −0.34; *p* = 0.02). This correlation was also visible in the positive correlation of the PAAT and the heart score and in the low to moderate correlation with the body mass (Table 7). The aortic blood flow profile was not significantly correlated to body mass and heart score in awake and anesthetized healthy racing pigeons (Table 7).

## 4. Discussion

The aim of this study was to examine the shape of the blood flow profile of the pulmonary artery and the aorta in 43 healthy racing pigeons. The detailed examination of the shape of the systolic Doppler sonographic blood flow profiles prepared by determining the percentage of the acceleration and deceleration time of the total blood flow period illustrates, in a better way, the differences between the blood flow profiles of the PA and AO in healthy racing pigeons [5,6]. The pulmonary Doppler sonographic blood flow profile shows a round shape, with the AP almost as long as DP, and in contrast to this, the systolic blood flow profile of the aorta is characterized by a shape with a short AP and a longer DP (Figure 1). These observations demonstrate the strong parallels between the relationships of the systolic blood flows of birds and small animals and humans [1,2,3,4]. The pressure conditions in the ventricles and in particular, the afterload of the ventricles, are mentioned as important factors for the development of the blood flow profiles [1,7,8,10,11]. In anesthetized pigeons, the examinations of the blood flow of the left ventricle and the aorta provided interesting insights into the pressure system of the pigeon heart. In the majority of the examined pigeons, the pressure in the left ventricle under anesthesia was too constant to cause a significant change in the shape of the AOFP. However, the positive correlation of the AP with the heart rate shows that the AP is prolonged at higher frequencies, which indicates a drop in pressure. Furthermore, there is a negative correlation of the heart rate with the systolic peak blood flow velocities, which supports the assumed pressure drop under anesthesia [6,21]. The literature shows that isoflurane depresses myocardial contractility and arterial elastance [22]. Thus, anesthesia with isoflurane also results in a decrease in the arterial blood pressure, cardiac output, and stroke volume in pigeons [22,23,24,25,26]. An increased heart rate under anesthesia is therefore considered an incipient anesthetic incident [6]. 

The behavior of the blood flow profile in the PA is difficult to interpret. In our study, a significant shortening of the AP was observed under anesthesia. This implies an increase in afterload, even though the significant negative influence of anesthesia on peak blood flow velocities in pigeons might demonstrate a drop in pressure [6]. This could be an indication that in pigeons, the blood flow profile of the PA is not exclusively determined by the blood pressure but also, for example, by the vascular resistance of the lungs, as in humans [8]. Dorsal recumbency, which is unfavorable for bird patients under anesthesia, but essential for sonographic examinations, and the associated increase in pressure on the lungs could be a possible explanation for this finding. Another influence on the PAFP may have been the altered arterial elastance during anesthesia with isoflurane, as described in mammals [22]. However, a prolongation of the AP of the PAFP, in conjunction with an increase in heart rate and a decrease in blood flow velocities in the PA in pigeons under anesthesia, confirm a drop in pressure [6].

The fact that the heart rate in awake pigeons was quite low, and that the heart rate was positively correlated with the AP of the PAFP in our examinations shows that the examination was not an extraordinarily stressful event for the pigeons. An additional explanation for the positive correlation of the heart rate and the AP of the PAFP could be a rapid adaptation of the lung circulation to an incipient increase in performance, which reduces the pressure load on the right heart in the short term. It will be interesting to observe the results of studies on other bird species in the future.

Blood flow velocities also provide insights into the pressure conditions of the heart, making the connection with the shape of the systolic blood flow profiles particularly interesting [5,6,21,27]. Specifically, the negative correlation of the peak flow velocities in AO and PA with the AP demonstrate that a higher presser influences the shape of the PAFP and AOFP. The significant negative correlation of the active left ventricular filling (A wave velocity, left) and the positive correlation of the E-to-A ratio, left, with the AP of the PAFP indicate the influence of the pressure of the left heart on the performance of the right heart. This relationship is particularly important in the development of right heart failure due to left heart failure, as described in human medicine [7,8,9,10,11,28], and also assumed in avian medicine [12,29,30].

Under anesthesia, the correlations of the E-to-A wave velocities of the left ventricle with the AOFP and of the A wave velocity of the right ventricle with the PAFP show the relationship between the diastolic blood flow and the systolic FP in pigeons. In pigeons, the A wave velocities in particular, as a mirror of the active filling pressure, are mainly influenced by active heart action [21]. These correlations also illustrate the different functions of the right and left ventricles; early filling appears to play a minor role in the function of the right ventricle in particular [21].

Our studies also demonstrate that body weight and heart-to-body size have an influence on the systolic blood flow profile. This shows that the pressure ratios in the heart can depend on body proportions, as an indication of breed-related differences in the pressure in the heart [31].

**Figure 2:**
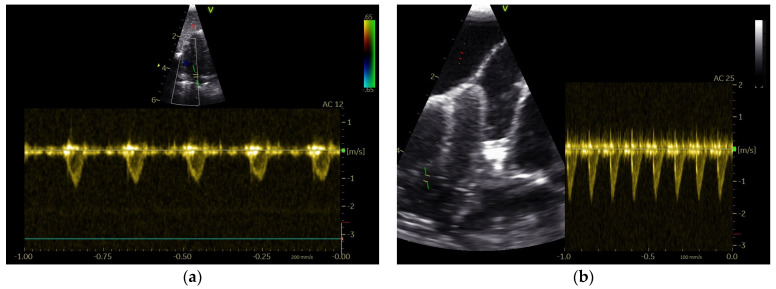
PW Doppler sonographic examination of the pulmonary blood flow profile of two Congo Grey parrots: (**a**) PAFP of a healthy Congo Grey parrot with a long AP and short DP; (**b**) PAFP of a Congo Grey parrot with left and right heart failure; the shape is similar to that of the AOFP, with a short AP and a long DP.

The various observed correlations provide an insight into the functioning of the pigeon heart. The significant physiological influences on the systolic blood flow profiles are proven in our examinations, but these are probably lower than those present in heart disease. Physiological influence and the influence of anesthesia did not lead to an overlap of the shape of the AOFP and PAFP in our investigations. Specifically, the PAFP profile seems to be affected by heart disease [12]. The generally lower pressure in the pulmonary circulation seems to be decisive for this observation [32,33]. A clinical case of a Congo Grey parrot with atherosclerosis illustrates how interesting the PAFP can be for the assessment of heart diseases. Figure 2 shows a Doppler sonographic examination of the PA of a Congo Grey parrot with left and right heart failure in comparison to the results for a healthy bird. The PAFP in the form of an AOFP demonstrates the higher pressure in the pulmonary circulation (pulmonary hypertension) due to left heart failure, as is also described in humans [28,34].

Some non-significant correlations were listed in this study because they can become relevant when there is more data available for comparison. This study included a relatively small trial group of only 43 pigeons, so some potentially interesting, but not significant, correlations may point to a lack of statistical power due to the small numbers.

Further studies need to determine whether the results for racing pigeons can be transferred to other bird species and whether the Doppler sonographic examination of the PAFP can be used to evaluate heart diseases in birds.

## 5. Conclusions

The AOFP differs significantly from the PAFP in awake as well as anesthetized pigeons. The AOFP is characterized by a short AP and a long DP. In contrast, the PAFP has a round appearance, with an AP similar to the DP. The low dependence on physiological parameters, such as heart rate, could also enable the use of Doppler sonographic PAFP for the assessment of heart diseases in birds. As the shape of the PAFP is similar to that of small mammals and humans, and because studies in small animal and human medicine show that diseases that lead to pulmonary hypertension have an influence on the shape of the PAFP, it can be assumed that the results will be similar in birds. This would make the measuring of the AP and DP a useful tool in further diagnosing heart failure in birds. More studies are needed using different bird species to determine the relevance of these findings throughout avian medicine.

## Figures and Tables

**Table 1:** Abbreviations of Doppler sonographic parameters used in this publication.

Abbreviation	Unit	Doppler Sonographic Parameter
AO	-	Aorta
PA	-	Pulmonary artery
AT	ms	Acceleration time
DT	ms	Deceleration time
ET	ms	Ejection time
AP	%	Percentage of AT on ET
DP	%	Percentage of DT on ET
PAAT	ms	AT of the blood flow of the PA
PADT	ms	DT of the blood flow of the PA
PAAP (%)	%	AP of the blood flow of the PA
PADP (%)	%	DP of the blood flow of the PA
PAET (ms)	ms	ET of the blood flow of the PA
PAV_max_ (m/s)	m/s	Peak flow velocities of the PA
E_RV_V_max_ (m/s)	m/s	Peak E wave velocities of the right heart
A_RV_V_max_ (m/s)	m/s	Peak A wave velocities of the right heart
E-to-A ratio _right_	-	E-to-A wave velocity ratio of the right heart
HR_PA_ (beats/min)	beats/min	Heart rate during measurements of the PA
AOAT (ms)	ms	AT of the blood flow of the AO
AODT (ms)	ms	DT of the blood flow of the AO
AOAP (%)	%	AP of the blood flow of the AO
AODP (%)	%	DP of the blood flow of the AO
AOET (ms)	ms	ET of the blood flow of the AO
AOV_max_ (m/s)	m/s	Peak flow velocities of the AO
E_LV_V_max_ (m/s)	m/s	Peak E wave velocities of the left heart
A_LV_V_max_ (m/s)	m/s	Peak A wave velocities of the left heart
E-to-A ratio _left_	-	E-to-A wave velocity ratio of the left heart
HR_AO_ (beats/min)	beats/min	Heart rate during measurement of the AO

**Table 2:** Doppler sonographic parameters of the awake pigeons examined in this study.

Parameter	Mean ± SD	Xmin–Xmax	Median	25% Percentile	75% Percentile
PAAT (ms)	38.1 ± 5.2	29.6–56.1	38.2	34.1	41.6
PADT (ms)	34.0 ± 7.5	12.1–53.0	34.2	28.7	38.2
PAAP (%)	53.3 ± 7.0	41.7–82.3	52.8	48.9	58.0
PADP (%)	46.7 ± 7.0	17.7–58.3	47.3	42.0	51.1
PAET (ms)	71.9 ± 8.3	53.6–92.4	71.5	67.2	77.1
PAV_max_	1.08 ± 0.20	0.70–1.59	1.06	0.93	1.21
E_RV_V_max_ (m/s)	0.22 ± 0.06	0.12–0.42	0.20	0.19	0.25
A_RV_V_max_ (m/s)	0.52 ± 0.09	0.31–0.72	0.51	0.45	0.60
E-to-A ratio, right	0.43 ± 0.11	0.25–0.71	0.40	0.36	0.50
HR_PA_ (beats/min)	207.5 ± 46.0	136.0–360.0	195.5	177.3	238.0
AOAT (ms)	18.9 ± 5.3	7.3–38.2	17.9	15.4	22.2
AODT (ms)	48.3 ± 6.2	38.5–72.7	47.4	44.7	50.8
AOAP (%)	28.1 ± 6.0	12.8–40.8	27.6	24.3	32.4
AODP (%)	71.9 ± 6.0	59.2–87.2	72.4	44.7	75.7
AOET (ms)	71.9 ± 8.3	53.6–92.4	71.5	67.2	77.1
AOV_max_ (m/s)	1.19 ± 0.15	0.79–1.55	1.19	1.08	1.28
E_LV_V_max_ (m/s)	0.37 ± 0.06	0.25–0.53	0.37	0.32	0.41
A_LV_V_max_ (m/s)	0.58 ± 0.20	0.24–0.96	0.57	0.42	0.72
E-to-A ratio, left	0.72 ± 0.28	0.31–1.63	0.64	0.50	0.91
HR_AO_ (beats/min)	209.8 ± 44.0	118.0–330.0	202.0	176.0	238.0

**Table 3:** Influence of the heart rate on the shape of the blood flow profile of the PA and AO in awake and anesthetized pigeons. The Spearman correlation coefficient r and the significance value *p* are shown. Significant correlations are marked in red. The green (*p* > 0.05 and ≤0.15) and blue (*p* > 0.15 and ≤0.25) colors show non-significant correlations.

Flow Profile Parameter	Heart Rate: Awake PA	Heart Rate: Awake AO	Heart Rate: Anesthetized PA	Heart Rate: Anesthetized AO
PAAT (ms)	*p* = 0.02 r = −0.30		*p* = 0.07 r = −0.27	
PADT (ms)	*p* = < 0.001 r = −0.55		*p* = 0.01 r = −0.41	
PAAP (%)	*p* = 0.02 r = 0.31		*p* = 0.15 r = 0.21	
PADP (%)	*p* = 0.02 r = −0.31		*p* = 0.15 r = −0.22	
AOAT (ms)		*p* = 0.05 r = −0.25		*p* = 0.24 r = −0.18
AODT (ms)		*p* = 0.01 r = −0.31		*p* = < 0.001 r = −0.52
AOAP (%)		*p* = 0.3 r = −0.13		*p* = 0.04 r = 0.3
AODP (%)		*p* = 0.3 r = 0.13		*p* = 0.04 r = −0.3

**Table 4:** Doppler sonographic parameters of the anesthetized pigeons examined in this study.

Parameters	Mean ± SD	Xmin–Xmax	Median	25% Percentile	75% Percentile
PAAT (ms)	51.3 ± 7.1	30.3–65.3	51.1	46.7	55.8
PADT (ms)	51.7 ± 11.5	31.8–81.3	52.2	42.5	58.5
PAAP (%)	50.2 ± 6.5	36.9–64.9	50.0	44.8	54.7
PADP (%)	49.8 ± 6.4	35.1–61.6	50.0	45.3	55.2
PAET (ms)	103.0 ± 13.9	78.3–146.6	102.0	96.3	110.9
PAV_max_ (m/s)	0.64 ± 0.11	0.40–0.88	0.64	0.56	0.71
E_RV_V_max_ (m/s)	0.18 ± 0.06	0.03–0.38	0.18	0.15	0.20
A_RV_V_max_ (m/s)	0.47 ± 0.11	0.24–0.80	0.45	0.41	0.53
E-to-A ratio _right_	0.42 ± 0.24	0.09–1.58	0.37	0.32	0.45
HR_PA_ (beats/min)	148.9 ± 30.6	98.0–248.0	149.0	131.0	162.0
AOAT (ms)	25.8 ± 5.2	15.7–38.0	25.3	22.2	29.3
AODT (ms)	64.5 ± 12.8	37.6–102.6	64.7	55.5	70.2
AOAP (%)	28.9 ± 5.3	18.7–39.6	28.3	24.7	32.4
AODP (%)	71.1 ± 5.3	60.4–81.3	71.7	67.6	75.3
AOET (ms)	90.3 ± 14.8	62.2–137.6	88.7	80.9	95.7
AOV_max_ (m/s)	0.95 ± 0.15	0.60–1.22	0.94	0.88	1.05
E_LV_V_max_ (m/s)	0.34 ± 0.07	0.26–0.52	0.33	0.29	0.38
A_LV_V_max_ (m/s)	0.42 ± 0.12	0.22–0.67	0.41	0.33	0.51
E-to-A ratio _left_	0.87 ± 0.32	0.44–1.79	0.78	0.62	1.14
HR_AO_ (beats/min)	154.6 ± 45.2	72.0–270.0	144.0	130.5	177.5

**Table 5:** Correlation of diastolic and systolic peak blood flow velocities with the shape of the blood flow profile of the PA and AO in **awake** pigeons. The Spearman correlation coefficient r and the significance value *p* are shown. Significant correlations are marked in red. The green (*p* > 0.05 and ≤0.15) and blue (*p* > 0.15 and ≤0.25) colors mark non-significant correlations.

FP Parameter	E_left_	A_left_	E-to-A_left_	E_right_	A_right_	E-to-A_right_	PA	Ao
PAAT (ms)	*p* = 0.32 r = 0.17	*p* = 0.5 r = −0.12	*p* = 0.28 r = 0.18	*p* = 0.91 r = 0.02	*p* = 0.62 r = −0.09	*p* = 0.9 r = 0.03	*p* = 0.03 r = −0.28	*p* = 0.43 r = −0.11
PADT (ms)	*p* = 0.37 r = 0.15	*p* = 0.08 r = 0.30	*p* = 0.21 r = −0.22	*p* = 0.09 r = 0.29	*p* = 0.34 r = 0.17	*p* = 0.9 r = 0.02	*p* = 0.001 r = −0.42	*p* = 0.42 r =- 0.11
PAAP (%)	*p* = 0.84 r = 0.03	*p* = 0.03 r = −0.37	*p* = 0.03 r = 0.37	*p* = 0.40 r = −0.14	*p* = 0.56 r = −0.10	*p* = 0.96 r = 0.01	*p* = 0.09 r = 0.22	*p* = 0.57 r = 0.08
PADP (%)	*p* =0.80 r = −0.04	*p* = 0.02 r = 0.37	*p* = 0.02 r = −0.38	*p* = 0.36 r = 0.16	*p* = 0.54 r = 0.10	*p* = 0.97 r = 0.01	*p* = 0.09 r = −0.22	*p* = 0.57 r = −0.08
AOAT (ms)	*p* = 0.85 r = 0.03	*p* = 0.17 r = 0.22	*p* = 0.07 r = −0.29	*p* = 0.38 r = −0.14	*p* = 0.4 r = −0.33	*p* = 0.26 r = −0.18	*p* = 0.82 r = −0.03	*p* = 0.29 r = 0.14
AODT (ms)	*p* = 0.25 r = −0.19	*p* = 0.36 r = 0.15	*p* = 0.21 r = −0.20	*p* = 0.79 r = 0.04	*p* = 0.96 r = 0.01	*p* = 0.9 r = −0.02	*p* = 0.08 r = −0.24	*p* = 0.98 r = 0.01
AOAP (%)	*p* = 0.76 r = 0.05	*p* = 0.44 r = 0.13	*p* = 0.25 r = −0.19	*p* = 0.32 r = −0.16	*p* = 0.60 r = −0.09	*p* = 0.37 r = −0.15	*p* = 0.78 r = 0.04	*p* = 0.56 r = 0.08
AODP (%)	*p* = 0.76 r = −0.05	*p* = 0.44 r = −0.13	*p* = 0.25 r = 0.19	*p* = 0.32 r = 0.16	*p* = 0.60 r = 0.09	*p* = 0.37 r = 0.15	*p* = 0.78 r = −0.04	*p* = 0.56 r = −0.08

**Table 6:** Correlation of diastolic and systolic peak blood flow velocities with the shape of the blood flow profile of the PA and AO in **anesthetized** pigeons. The Spearman correlation coefficient r and the significance value *p* are shown. Significant correlations are marked in red. The green (*p* > 0.05 and ≤0.15) and blue (*p* > 0.15 and ≤0.25) colors mark non-significant correlations.

FP Parameter	E_left_	A_left_	E-to-A_left_	E_right_	A_right_	E-to-A_right_	PA	Ao
PAAT (ms)	*p* = 0.21 r = 0.23	*p* = 0.08 r = −0.32	*p* = 0.05 r = 0.35	*p* = 0.25 r = −0.22	*p* = 0.6 r = −0.1	*p* = 0.66 r = 0.09	*p* = 0.15 r = −0.22	*p* = 0.72 r = 0.06
PADT (ms)	*p* = 0.21 r = 0.23	*p* = 0.67 r = 0.08	*p* = 0.93 r = 0.17	*p* = 0.14 r = 0.28	*p* = 0.03 r = 0.40	*p* = 0.42 r = −0.16	*p* = 0.12 r = 0.23	*p* = 0.71 r = −0.07
PAAP (%)	*p* = 0.65 r = −0.08	*p* = 0.22 r = −0.23	*p* = 0.46 r = 0.14	*p* = 0.09 r = −0.32	*p* = 0.13 r = −0.27	*p* = 0.59 r = 0.11	*p* = 0.03 r = −0.32	*p* = 0.82 r = 0.04
PADP (%)	*p* = 0.64 r = 0.09	*p* = 0.22 r = 0.23	*p* = 0.46 r = −0.14	*p* = 0.10 r = 0.31	*p* = 0.11 r = 0.28	*p* = 0.55 r = −0.12	*p* = 0.03 r = 0.32	*p* = 0.80 r = −0.04
AOAT (ms)	*p* = 0.1 r = 0.26	*p* = 0.23 r = −0.19	*p* = 0.11 r = 0.26	*p* = 0.91 r = −0.02	*p* = 0.29 r = 0.17	*p* = 0.63 r = −0.08	*p* = 0.33 r = 0.18	*p* = 0.69 r = 0.06
AODT (ms)	*p* = 0.03 r = 0.33	*p* = 0.55 r = −0.1	*p* = 0.03 r = 0.33	*p* = 0.43 r = 0.13	*p* = 0.08 r = 0.27	*p* = 0.45 r = −0.13	*p* = 0.82 r = −0.04	*p* = 0.01 r = 0.38
AOAP (%)	*p* = 0.57 r = −0.09	*p* = 0.85 r = −0.03	*p* = 0.53 r = −0.10	*p* = 0.47 r = −0.12	*p* = 0.79 r = −0.04	*p* = 0.95 r = −0.01	*p* = 0.45 r = 0.13	*p* = 0.06 r = −0.28
AODP (%)	*p* = 0.57 r = 0.09	*p* = 0.85 r = 0.03	*p* = 0.53 r = 0.10	*p* = 0.47 r = 0.12	*p* = 0.79 r = 0.04	*p* = 0.95 r = 0.01	*p* = 0.45 r = −0.13	*p* = 0.06 r =0.28

**Table 7:** Correlation of body mass and heart score with the shape of the blood flow profile of the PA and AO in **awake** and **anesthetized** pigeons. The Spearman correlation coefficient r and the significance value *p* are shown. Significant correlations are marked in red. The green (*p* > 0.05 and ≤0.15) and blue (*p* > 0.15 and ≤0.25) colors mark non-significant correlations.

	Awake Pigeons		Anesthetized Pigeons	
FP Parameters	Body Mass	Heart Score	Body Mass	Heart Score
PAAT (ms)	*p* = 0.22 r = 0.16	*p* = 0.03 r = 0.29	*p* = 0.04 r = 0.32	*p* = 0.15 r = 0.22
PADT (ms)	*p* = 0.99 r = 0.01	*p* = 0.32 r = 0.13	*p* = 0.23 r = −0.18	*p* = 0.13 r = −0.23
PAAP (%)	*p* = 0.54 r = 0.08	*p* = 0.02 r = 0.33	*p* = 0.03 r = 0.33	*p* = 0.02 r = 0.33
PADP (%)	*p* = 0.56 r = −0.08	*p* = 0.02 r = −0.34	*p* = 0.03 r = −0.32	*p* = 0.02 r = −0.34
AOAT (ms)	*p* = 0.23 r = 0.15	*p* = 0.60 r = −0.68	*p* = 0.32 r = −0.16	*p* = 0.45 r = −0.12
AODT (ms)	*p* = 0.63 r = −0.06	*p* = 0.61 r = −0.07	*p* = 0.26 r = −0.17	*p* = 0.91 r = −0.02
AOAP (%)	*p* = 0.10 r = 0.21	*p* = 0.7 r = −0.05	*p* = 0.78 r = 0.04	*p* = 0.58 r = −0.08
AODP (%)	*p* = 0.10 r = −0.21	*p* = 0.70 r = 0.05	*p* = 0.78 r = −0.04	*p* = 0.58 r = 0.08

## Data Availability

The data from this study are included in the text.
